# Remarks on the Comparative Value of the Defferent Anæsthetic Agents

**Published:** 1850-06

**Authors:** 


### ECLECTIC DEPARTMENT,

### AND SPIRIT OF THE MEDICAL PERIODICAL PRESS.

---

*Remarks on the Comparative Value of the defferent Anæsthetic Agents.* By George Haywrad, M. D., one of the surgeons to the Massachusetts General Hospital.

This is the title of a small pamphlet republished from the Boston Medical and Surgical Journal. We transfer to our columns all that relates to the subject practically.

1*st. Of Sulphuric Ether.*

When sulphuric ether was first administered by inhalation, it was by means of a pretty formidable-looking and expensive apparatus. Various instruments for this purpose were constructed, both in this country and Europe. The same objections applied to all of them. They were so formed as to create a well-founded apprehension that the supply of atmospheric air would not in every case be sufficient. It was difficult to guard against this; and from this cause, some patients, soon after the discovery was made, nearly lost their lives by asphyxia.

Besides, to use them with entire success required, in a greater or less degree, the coöperation of the individual to whom the ether was administered. This of course could not always be had, and the consequence was that very frequently a sufficient degree of insensibility was not produced, and even when it was, it could not be kept up as long as in many cases was desirable.

The cost of the apparatus, too, was a serious objection, though a vastly less important one than either of the others that I have named. At the same time it was so great, that if some simpler and less expensive mode of administering either had not been found, it may well be doubted whether the benefits of the discovery would have been as rapidly and extensively diffused as they have been.

But all these objections are entirely obviated by the use of a bell-shaped sponge of fine texture. This should be large enough to cover the nose and mouth. The patient is required to do nothing. The apparatus is simple and not costly.

This mode was adopted at the Massachusetts Hospital in a few months after the first use of ether there by inhalation; I am not aware that it was previously used anywhere else, and I presume that it is now the only method by which ether is inhaled.

The quantity necessary to produce the desired effect must vary in different cases. In surgical operations, requiring from five to ten minutes for their performance, from three to six ounces is usually sufficient. The ether, however, should be of the purest kind, that is the rectified, which has undergone a second distillation, by means of which it parts with a considerable portion of its alcohol. Yet a much greater quantity than what has been named can be used with perfect safety, and the patient may be kept for a much longer time under its influence without danger, by occasionally removing the sponge, and re-applying it when he gives signs of returning sensibility.

By administering it gradually, many unpleasant effects are avoided. The great irritation of the larynx and air passages, accompanied by urgent and convulsive cough, is in most cases entirely prevented. The vapor of the ether should be so mixed with atmospheric air, that respiration should be neither laborious nor painful. The irritability of the parts with which the ether comes in contact is by degrees overcome, and then the sponge may be applied directly to the face, and if uecessary compressed in some measure so as to exclude to a greater degree the atmospheric air. When the desired effect is produced, which is usually in from three to five minutes, the patient has no control over the voluntary muscles; he cannot speak; he cannot open his eyes, when directed to do so; his muscles become completely relaxed, and the pulse, which at the beginning of the inhalation is frequent and often rises during the process to 140 beats in a minute or more, become slower, and I have very often known it to fall to 60. The patient is then insensible and unconscious, and the surgeon may begin his operation with great confidence that he will inflict no suffering. The sponge should then be removed, and reapplied from time to time as circumstances may require. If the ether is not pure, longer time is necessary to produce the desired effect; the brain and nervous system are more excited, and the patient is occasionally violent for a time and with difficulty controlled.

Before using the ether the sponge should be dipped in warm water, and then strongly compressed, leaving it slightly damp. The evaporation seems to go on better in this way than when a sponge is used that has not been previously moistened. In the first instance, the ether should be poured on the inside of the sponge; about two ounces is enough; when more is required, it should be applied to the outside, as it is best not to remove the sponge from the face.

Sulphuric ether of a proper quality used in this way, I am confident, is perfectly safe, and will in almost every instance produce the desired effect. I have administered it to persons of all ages, of every variety of constitution, and in almost every state of the system, and I have never known in a single instance a fatal or alarming result. I have given it to infants of seven weeks old, and to individuals of 75 years, with entire success. I have administered it to persons suffering under chronic pulmonary disease, not only without injury, but in some cases with decided benefit. It is well known that it often gives relief in catarrhal affections of the lungs and in paroxysms of asthma. In fact, I hardly know a state of the system in which I should be deterred from using it, if I were called upon to perform a surgical operation.

The advantages, then, of sulphuric ether as an anæsthetic agent, are its entire safety, the ease with which it is administered, and the slight inconvenience which follows its administration. I have already stated that I have never known its inhalation followed by a fatal or alarming effect, and there is reason to doubt whether death has in a single instance been produced by it, when it has been properly administered. One patient is said to have lost his life by its inhalation at the Hospital in Auxerre, in France. This took place in August, 1847. The details of the case are not given with such minuteness as to enable any one to form a satisfactory opinion. It occurred, however, not long after the discovery; before the best mode of exhibiting it was adopted, and the *post-mortem* appearances indicated, as far as any opinion could be formed from them, that death was caused by asphyxia. In a careful examination of some of the leading medical journals of Europe and this country, published during the last three years, I have not been able to find another case in which life was destroyed by the inhalation of sulphuric ether, and there is reason to believe, as I have already intimated, that death would not have taken place in this instance, if the lungs had been abundantly supplied with atmospheric air. It is only wonderful that an agent of such power, used as it often has been in the most reckless manner, by unskilful and ignorant persons, should not have caused far more disastrous results, than any that have hitherto been made known. It teaches us that though it should be used with caution and confided only to skilful hands, the dangers from its use are far less than our preconceived opinions had led us to believe.

The great ease with which it can be administered is not to be overlooked in estimating its advantages. No complicated apparatus is required, and no coöperation of the patient is necessary. A simple sponge, moistened with sulphuric ether and held before the face for two or three minutes, will in almost every instance produce the desired effect.

There are no ill consequences from its use. If it be breathed only for a short time, its effects usually pass off in a few minutes. I have never known them to continue for more than an hour; and in this case the patient had been kept under its influence for forty-five minutes. Nausea and vomiting are not frequent, unless it is inhaled soon after food has been taken. I have not seen convulsions follow its exhibition, nor any delirium, except a slight and transitory kind, such as arises from intoxicating liquors. I confess that I was much surprised to learn, by carefully watching its effects, to what a small extent and for how short a time it disturbed the functions of the nervous system, and how rare it was to find headache among the consequences of its inhalation.

If, however, the state of narcotism should continue longer than is necessary for the purposes for which it was produced, the means that seem to me the most likely to remove it, are the dashing of cold water in the face; the application of strong stimulants, as the carbonate of ammonia, to the nose; and, as soon as the patient can swallow, the administration of a small quantity of hot spirit and water. The object is to increase the action of the heart, so that the blood may circulate more rapidly through the lungs, and thus be enabled to part with the vapor of the ether that is mixed with it. When narcotism arises from any noxious substance taken into the stomach, we adopt means to empty that organ as soon as possible by the stomach pump or an emetic. The principle of the treatment in the two cases is the same; the object being in both to remove the cause of the peculiar state of the system under which the patient is laboring.

The only objections of which I am aware to sulphuric ether as an anæsthetic agent, are its pungent odor, which is offensive to some persons, and the no inconsiderable degree of irritation which its inhalation occasionally produces in the air passages. This irritation, I am confident, may be in great measure prevented by proper attention to the mode of its exhibition and the quality of the article used. Admitting these objections to be as great as they have been said to be by those who have urged them with the most earnestness, they do not in my opinion counterbalance the advantages; and I have no hesitation in saying that I should give it the preference over any other article with which I am acquainted, that is used for the purpose of producing insensibility.

2d. *Of Chloroform.*

Chloroform is the perchlorid of formyle, the radicle of formic acid. It has been ascertained by Dumas to consist of three parts of chlorine to one of the bi-carburet of hydrogen [formyle]. It was discovered almost simultaneously nearly twenty years since in France, Germany, and this country.

It was first employed as an anæsthetic agent by Professor Simpson, of Edinburgh. and he thought that it possessed "various important advantages" over sulphuric ether. He says that "it is far more portable; more manageable and powerful; more agreeable to inhale; is less exciting than ether; and gives us far greater control and command over the superinduction of the anæsthetic state." If all this were true, it would no doubt be preferable to any other agent with which we are acquainted. But subsequent experience proves that it is not so.

Its only advantages are that it is more agreeable to inhale than ether, and that a less quantity of it answers the purpose. On the other hand, it cannot be denied that fatal effects have followed its inhalation in several instances even when administered by the most judicious hands; that in some cases convulsions have been produced, and in others a great disturbance of the brain causing delirium. In some persons this affection of the mind has continued for several weeks.

There are other objections of a minor character. Chloroform is of an acrid, caustic nature, and if it come in contact with the skin, unless it be protected by some oily substance, severe excoriation is the consequence. Its administration is generally followed by vomiting and headache, which continues for several hours, attended by a great degree of restlessness and want of sleep. Several cases have come under my care, in which the brain and nervous system have been affected to an alarming extent; though in every instance, it was said that a small quantity only of chloroform was administered for the purpose of performing some operation on the teeth.

An individual in this vicinity was thrown into violent convulsions, which continued for three or four days, during all which time she was in a state of complete insensibility, from the inhalation of the vapor of a few drops of chloroform administered by a careful and judicious physician. It would be easy to multiply examples of this kind; but it is not necessary, for there is a stronger ground on which we can rest our opposition to the use of chloroform, that is, its danger to life. This, it is well known, has already been in several instances destroyed by it. If it can be shown that it has caused the death of a single individual, when properly administered, we cannot fail to have our misgivings of the safety of its exhibition, though it may have been inhaled in almost numberless cases without any ill effect.

I am satisfied that there are already on record at least twenty well-authenticated cases of death from the inhalation of chloroform; and I know not how a conscientious man, knowing this fact, can willingly take the responsibility and expose his patient to this fearful result. One of the conclusions to which M. Malgaigne arrives, in his report on chloroform, to the Academy of Medicine of Paris, cannot be too strongly impressed on the minds of those who feel inclined to use it. "Chloroform possesses a toxic action peculiar to itself, which has been taken advantage of in medicine by arresting it at the period of insensibility, which action, however, may, by being too much prolonged, cause immediate death." The danger is that we cannot always know the precise time to arrest it, and that the fatal blow may be struck before we make the attempt. In other words, chloroform is a poison, and the insensibility which it produces is only the first stage of its poisonous action.

3d. *Of Chloric Ether.*

There are two kinds of chloric ether. The one, the strong or concentrated; and the other, the chloric ether of commerce. They are both tinctures of chloroform, differing from each other only in the relative proportions of the alcohol and chloroform of which they are composed. The concentrated consists of one part of chloroform to nine parts of alcohol; and in the chloric ether of commerce, there is one part of chloroform to fifteen of alcohol. The former is the one that is sometimes used for inhalation.

It is said to have been first recommended for this purpose by one of the most eminent surgeons of Great Britain, William Lawrence, Esq., of London; but I cannot learn that it is now employed in Europe to any extent in this way. In fact, it is hardly spoken of at all in the foreign medical journals that I have seen. and I have examined a large number with this view. It has been tried, however, pretty extensively by Dr. J. C. Warren and Dr. J. Mason Warren, both at the Hospital and in private practice, and I am not aware that any ill effects have followed its use. On the contrary, I believe that they are well satisfied with it, and prefer it to the other anæsthetic agents.

At the same time it cannot be denied that it derives its power of producing insensibility from the chloroform it contains; and it is difficult to understand how the addition of alcohol can deprive it of its dangerous properties, when it well known that the mixture of this substance with sulphuric ether renders it in a great measure unfit for inhalation.

The advantages which it is said to possess are, that its odor is less pungent and disagreeable than that of sulphuric ether, and that it can be inhaled with little or no inconvenience. At the same time it must be admitted that it is necessary to use as much chloric as sulphuric ether, and to continue the inhalation for as long a time to produce the desired effect.

The disadvantages are, that when it comes in contact with the unprotected skin it acts upon it in the same manner as chloroform. From this cause a patient suffered several months at the Hospital, and I believe much more severely than if he had undergone the operation without the ether. I am confident, too, that it is more apt to produce vomiting, and a greater disturbance of the brain and nervous system, causing headache, restlessness and vigilance, which not unfrequently continue for many hours after its exhibition. Perhaps these last symptoms may be owing to the great amount of alcohol it contained.

I cannot, I confess, divest myself of the belief that chloric ether is an unsafe anæsthetic agent, when I consider that it is simply chloroform diluted with alcohol. It is true, that as far as we know, no fatal effects have hitherto followed its inhalation; but it is also true, that it has as yet been used to a very limited extent, and in all the cases in which it has been exhibited that have come to my knowledge, it has been managed with great caution and judgment. But I fear that if it be used with the same freedom that sulphuric ether is, we shall soon have to record some very different results. We cannot feel confident that it will always be confided to skilful hands only, nor by any means certain that death, when not looked for, may not follow its inhalation.

*Boston, April* 10, 1850.

